# Novel porcine-like human G26P[19] rotavirus identified in hospitalized paediatric diarrhoea patients in Ho Chi Minh City, Vietnam

**DOI:** 10.1099/vir.0.068403-0

**Published:** 2014-12

**Authors:** Phan Vu Tra My, Maia A. Rabaa, Celeste Donato, Daniel Cowley, Voong Vinh Phat, Tran Thi Ngoc Dung, Pham Hong Anh, Ha Vinh, Juliet E. Bryant, Paul Kellam, Guy Thwaites, Mark E. J. Woolhouse, Carl D. Kirkwood, Stephen Baker

**Affiliations:** 1 The Hospital for Tropical Diseases, Wellcome Trust Major Overseas Programme, Oxford University Clinical Research Unit, Ho Chi Minh City, Vietnam; 2 The Wellcome Trust Sanger Institute, Hinxton, Cambridge, UK; 3 Centre for Immunity, Infection and Evolution, University of Edinburgh, Edinburgh, UK; 4 Murdoch Childrens Research Institute, Melbourne, Australia; 5 La Trobe University, Melbourne, Australia; 6 The Hospital for Tropical Diseases, Ho Chi Minh City, Vietnam; 7 Wellcome Trust Major Overseas Programme, Oxford University Clinical Research Unit, Hanoi, Vietnam; 8 Division of Infection and Immunity, University College London, London, United Kingdom; 9 Centre for Tropical Medicine, Nuffield Department of Clinical Medicine, Oxford University, Oxford, UK; 10 The London School of Hygiene and Tropical Medicine, London, UK

## Abstract

During a hospital-based diarrhoeal disease study conducted in Ho Chi Minh City, Vietnam from 2009 to 2010, we identified four symptomatic children infected with G26P[19] rotavirus (RV) – an atypical variant that has not previously been reported in human gastroenteritis. To determine the genetic structure and investigate the origin of this G26P[19] strain, the whole genome of a representative example was characterized, revealing a novel genome constellation: G26–P[19]–I5–R1–C1–M1–A8–N1–T1–E1–H1. The genome segments were most closely related to porcine (VP7, VP4, VP6 and NSP1) and Wa-like porcine RVs (VP1–3 and NSP2–5). We proposed that this G26P[19] strain was the product of zoonotic transmission coupled with one or more reassortment events occurring in human and/or animal reservoirs. The identification of such strains has potential implications for vaccine efficacy in south-east Asia, and outlines the utility of whole-genome sequencing for studying RV diversity and zoonotic potential during disease surveillance.

Group A rotaviruses (RVs) are ubiquitous and are the principal cause of acute gastroenteritis in children. RV is highly transmissible and it is estimated that nearly every child in the world is infected before reaching 5 years of age (Bernstein, 2009; Cortese *et al.*, 2009; Glass *et al.*, 2006; Parashar *et al.*, 2003, 2006; Tate *et al.*, 2012). RVs are non-enveloped dsRNA viruses with a genome consisting of 11 independent gene segments, encoding six structural proteins (VP1–4, VP6 and VP7) and six non-structural proteins (NSP1–NSP5/6) (Estes *et al.*, 2007). Molecular characterization of the outer capsid proteins, VP7 and VP4, differentiates strains into the various G and P genotypes (Estes *et al.*, 2007). Classification of RV using all 11 genomic segments has recently been adopted, resulting in a strain whole-genome nomenclature of G*x*–P[*x*]–I*x*–R*x*–C*x*–M*x*–A*x*–N*x*–T*x*–E*x*–H*x*, representing the genotypes of VP7–VP4–VP6–VP1–VP2–VP3–NSP1–NSP2–NSP3–NSP4–NSP5, respectively (Matthijnssens *et al.*, 2011). To date, 27 G, 37 P, 17 I, 9 R, 9 C, 8 M, 18 A, 10 N, 12 T, 15 E and 11 H types have been identified in humans and non-human hosts (Abe *et al.*, 2011; Guo *et al.*, 2012; Jere *et al.*, 2014; Matthijnssens *et al.*, 2011; Papp *et al.*, 2012; Trojnar *et al.*, 2013).

We conducted a prospective multicentre hospital-based study of acute diarrhoea in children during 2009–2010 in Ho Chi Minh City (HCMC), Vietnam. This study was conducted according to the principles expressed in the Declaration of Helsinki, and was approved by the institutional ethical review boards of all participating hospitals and the Oxford Tropical Research Ethics Committee (My *et al.*, 2013). Stool samples from symptomatic and asymptomatic children were collected and screened, as described previously, for multiple aetiological agents, including parasites by microscopy, bacteria (*Shigella*, *Salmonella*, *Campylobacter*, *Yersinia*, *Pleisiomonas* and *Aeromonas*) by microbiological culture and viruses (norovirus and RV) by reverse transcription (RT)-PCR amplification (My *et al.*, 2013). For RV identification and genotyping, RT-PCR was performed on total dsRNA from stool samples to detect the RV capsid genes, VP7 and VP4, followed by direct sequencing of the PCR amplicons for confirmation and genotyping (My *et al.*, 2011). G and P types were determined via the group A RV genotyping tool, RotaC v2.0 (http://rotac.regatools.be), according to the guidelines of the Rotavirus Classification Working Group (Maes *et al.*, 2009).

Over the study period (from May 2009 to April 2010), RV was detected in the stool samples of 664 of 1419 (46.8 %) children with acute diarrhoea, of which 590 (88.4 %) patients were infected with RV only, and none of the other diarrhoeal pathogens screened for were identified. Sequence analysis of the RV-positive samples revealed a diverse array of genotypes, including a novel G26P[19] genotype in four children with symptomatic disease. The ages of the four patients infected with a G26P[19] strain ranged from 14 to 24 months (Table S1, available in the online Supplementary Material), slightly higher than the median age of RV infection caused by other genotypes (13 months; interquartile range: 9–19 months). The geographical and temporal distribution of the four G26P[19] infections suggested that these cases were not associated epidemiologically, making it difficult to determine whether some or all of these cases resulted from a human-to-human transmission chain or represented multiple jumps from non-human hosts to humans (Table S1). We found none of the other screened diarrhoeal pathogens in the stool samples of these four children and therefore assumed that G26P[19] RV was the causative agent of diarrhoeal episodes observed in all cases. These G26P[19] RV infections were detected at locations across HCMC at different time points throughout enrolment, which suggested that additional undetected G26P[19] infections may have occurred in the broader population during this period. As this study was hospital-based, milder G26P[19] infections in the community may have gone unreported or presented to alternative healthcare facilities in the city. Furthermore, as sampling was conducted over a period of 1 year, we were unable to determine whether this atypical genotype continued to circulate locally after the period of investigation.

In accordance with recent changes in RV classification (Matthijnssens *et al.*, 2011) and to investigate the potential origins of the strain, we characterized all 11 gene segments of a representative G26P[19] strain to determine its genome constellation. Genome sequencing was performed on the strain RVA/Human-wt/VNM/30378/2009/G26P[19], using conventional Sanger sequencing after a series of combinatorial RT-PCR amplifications with an array of internal primers (Table S2) using a previously described approach (Cowley *et al.*, 2013). The coding region for the 11 gene segments was determined and the resulting genome constellation was determined to be G26–P[19]–I5–R1–C1–M1–A8–N1–T1–E1–H1 (GenBank accession numbers HG513045–HG513055) – a novel combination of genome segments that has not been reported previously. The genome constellation of the sequenced strain was compared to other sequences of selected animal and human strains in GenBank, including representative strains containing G26 and P[19] segments (Tables 1 and S3).

**Table 1.** The genotypes of 11 segments of RVA/Human-wt/VNM/30378/2009/G26P[19] compared to those of selected human and animal RV strains of known genotypes

Strain	Genotypes										
VP7	VP4	VP6	VP1	VP2	VP3	NSP1	NSP2	NSP3	NSP4	NSP5
RVA/Human-wt/VNM/30378/2009/G26P[19]	*G26*	*P[19]*	*I5*	*R1*	*C1*	*M1*	*A8*	*N1*	*T1*	*E1*	*H1*
RVA/Pig-wt/JPN/TJ4-1/2010/G26P[7]	*G26*	P[7]	–	–	–	–	–	–	–	–	–
RVA/Human-wt/THA/57vp7w/200X/G3P[19]	G3*	*P[19]*	–	–	–	–	–	–	–	–	–
RVA/Human-tc/THA/Mc323/1989/G9P[19]†	G9	*P[19]*	*I5*	***R1***	*C1*	*M1*	***A8***	*N1*	*T1*	*E1*	*H1*
RVA/Human-tc/THA/Mc345/1989/G9P[19]†	G9	*P[19]*	*I5*	*R1*	*C1*	*M1*	*A8*	*N1*	***T1***	*E1*	*H1*
RVA/Human-wt/IND/NIV929893/1992/G1P[19]†	G1	***P[19]***	I1	–	–	–	–	–	–	*E1*	–
RVA/Pig-wt/ARG/CN86/1988/GXP[X]	–	–	**I5**	–	–	–	–	–	–	–	H1
RVA/Pig-wt/THA/CMP45/08/2008/G9P[23]	G9	P[23]	I5	*R1*	***C1***	*M1*	*A8*	*N1*	T7	*E1*	*H1*
RVA/Human-wt/HUN/BP1490/1994/G4P[6]†	G4	P[6]	I1	*R1*	*C1*	***M1***	*A1*	*N1*	T7	*E1*	***H1***
RVA/Pig-wt/CHN/LLP48/2008/G9P[6]	G9	P[6]	I1					*N1*		***E1***	
RVA/Human-wt/BEL/B3458/2003/G9P[8]†	G9	P[8]	I1	*R1*	*C1*	*M1*	A1	***N1***	*T1*	*E1*	*H1*

Italic indicates gene segments with a genotype identical to that of strain RVA/Human-wt/VNM/30378/2009/G26P[19]. Bold indicates segments with the highest nucleotide sequence identities to that of strain RVA/Human-wt/VNM/30378/2009/G26P[19]. A dash (–) indicates the unavailability of sequence data in GenBank.

* This strain was identified as a rare G3P[19] genotype (Theamboonlers *et al.*, 2008), yet appeared to be a G26P[19] genotype based on the RotaC genotyping tool and the nucleotide sequence identity to that of our G26P[19] strain.

† Strains shown previously to have a porcine genetic backbone and porcine origin.

To investigate segment-specific evolutionary relationships for the sequenced G26P[19] strain, maximum-likelihood phylogenies were inferred for each of the 11 gene nucleotide sequences and related reference RV sequences obtained from GenBank. Multiple alignments of nucleotide sequences and amino acid sequences were performed using mega5 employing clustal w algorithms, and were visually checked and realigned using Se-AL (v2.0) (http://tree.bio.ed.ac.uk/software/seal/). Only complete gene sequences were utilized in phylogenetic analyses, with the exception of VP4 [which included all Vietnamese P[19] sequences from this study regardless of sequence length (minimum sequence length: 619 nt) and RVA/Human-wt/THA/57vp7w/200X/G3P[19]] and VP7 [which included all Vietnamese G26 sequences from this study (minimum sequence length: 821 nt) and any available G26 sequences available in GenBank regardless of length]. Phylogenetic trees were inferred for each gene segment using aligned nucleotide sequences, employing a maximum-likelihood method in PhyML v3.0 (Guindon *et al.*, 2010), using the HKY85 model of substitution (which was determined to be the best fit model for all gene segments using modeltest; Posada & Crandall, 1998) with 1000 bootstrap replicates. The resulting phylogenetic trees were visualized and edited in FigTree (v1.4) (http://tree.bio.ed.ac.uk/software/figtree/) and genetic distances (*p*-uncorrected) were estimated using HyPhy v2.0 (http://www.datam0nk3y.org/hyphy/doku.php) (Pond *et al.*, 2005).

Phylogenetic analysis of VP7 was performed on all available global G26 sequences and additional sequences from the three G26P[19] strains identified in this study (GenBank accession numbers HG513056–HG513058). The VP7 sequences of the G26 strains fell within a single distinct lineage (Fig. 1), with the VP7 gene of RVA/Human-wt/VNM/30378/2009/G26P[19] being most closely related to the equivalent gene in the G26 strain RVA/Human-wt/THA/57vp7w/200X/G3P[19] from Thailand (incorrectly labelled as G3 in GenBank) (Theamboonlers *et al.*, 2008) compared with the porcine G26 strains RVA/Pig-wt/JPN/TJ4-1/2010/G26P[7] (Miyazaki *et al.*, 2011) and RVA/Pig-wt/CHN/Z650/2007/G26P[X] (GenBank accession number KC292205; unpublished). Calculations of *p*-uncorrected genetic distance also showed the highest similarity between the VP7 sequence of our strain and that of the 57vp7w strain (97.1 %; 736 bp), followed by the corresponding segments of TJ4-1 (93.2 %; 987 bp) and Z650 (92.8 %; 1061 bp) strains (Tables 1 and S3). All of the VP7 segments from the G26 strains identified in human hosts fell within a sublineage of the G26 genotype that was closely related to those isolated from porcine hosts (Fig. 1). Although these analyses were limited by the paucity of available full-length RV genome sequences from human and animal species, phylogenetic clustering suggested that viruses of this novel genotype may have moved between porcine and human hosts on at least one occasion, followed by limited onward transmission in humans (Fig. 1).

**Fig. 1.**
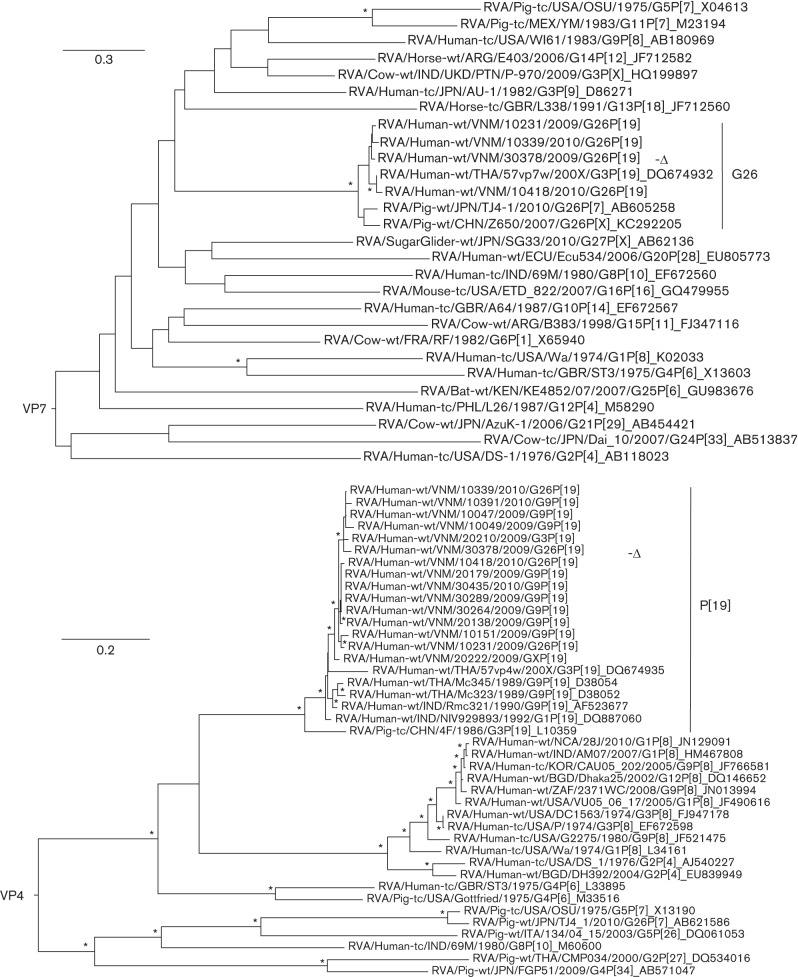
Phylogenetic trees inferred from nucleotide sequences for VP7 and VP4 gene segments of our G26P[19] strain with the corresponding segments of other group A RV strains. Maximum-likelihood trees of VP7 and VP4 (partial ORFs) genes show relationships between these genes in RVA/Human-wt/VNM/30378/2009/G26P[19] and additional sequences from GenBank. The strain RVA/Human-wt/VNM/30378/2009/G26P[19] is indicated by ‘-Δ’ throughout. Trees are midpoint rooted for the purpose of clarity and asterisks indicate bootstrap values ≥80 %. All horizontal branch lengths are drawn to the scale of nucleotide substitutions per site in each tree.

Our strain, RVA/Human-wt/VNM/30378/2009/G26P[19], contained the P[19] VP4 gene segment (Fig. 1). Like G26, P[19] is also reported infrequently and has only been detected in pigs and a limited number of symptomatic humans in South and East Asia. Shortly after its initial identification in pigs (RVA/Pig-tc/CHN/4F/1986/G3P[19]) in China (Burke *et al.*, 1994), P[19] segments were identified in RV strains isolated from human infections in India (G9P[19]) (Okada *et al.*, 2000). Cumulative evidence from genetic investigations and the sustained detection of P[19] in humans and pigs support the theory that P[19] may have originated in pigs, suggesting that P[19] was introduced into human RV strains via human–porcine RV reassortment (Burke *et al.*, 1994; Ghosh *et al.*, 2012; Krishnan *et al.*, 1994; Maneekarn *et al.*, 2006; Mukherjee *et al.*, 2011; Nguyen *et al.*, 2008; Okada *et al.*, 2000; Theamboonlers *et al.*, 2008; Urasawa *et al.*, 1992; Varghese *et al.*, 2004; Wu *et al.*, 2011; Zade *et al.*, 2009). The complete nucleotide sequence of the VP4 gene of our RVA/Human-wt/VNM/30378/2009/G26P[19] demonstrated the highest *p*-uncorrected nucleotide sequence similarity (95.7 %) with the porcine–human reassortant strain RVA/Human-wt/IND/NIV929893/1992/G1P[19] (Chitambar *et al.*, 2009) (Table 1), and clustered with other full-length porcine and porcine-like human P[19] sequences in the VP4 phylogeny (Fig. 1). A RV strain incorporating a P[19] VP4 segment was reported previously in a symptomatic RV patient in HCMC in 2003 (Nguyen *et al.*, 2008); the resulting P[19] sequence for this RV strain (RVA/Human-wt/VNM/VN375/2003/GXP[19]) was only 558 bp in length, but exhibited 95.7 % nucleotide sequence identity to our more recently sampled viruses. These findings raise a question as to whether RV strains containing P[19] represent an established but rarely detected variant circulating in humans in Vietnam or are a porcine-maintained genotype that has undergone repeated jumps into the human population.

The remaining genes encoding the structural proteins (VP6 and VP1–3) exhibited the highest nucleotide sequence identity to the corresponding genes of porcine strains or porcine–human reassortants. The VP6 full-length sequence of our G26P[19] exhibited a sequence identity of 91.1 % to the cognate genes of the porcine strain RVA/Pig-wt/ARG/CN86/1988/GXP[X] (Table 1), and clustered phylogenetically with other porcine, porcine-like human and porcine-like non-human strains (Fig. S1). Additionally, the VP1–3 sequences of our G26P[19] strain clustered phylogenetically with the cognate segments of porcine strains and (porcine-like) human strains (Fig. S1), and demonstrated the highest sequence similarity to porcine and porcine-like human strains (Tables 1 and S3). Similarly, further genetic distance determination and phylogenetic analyses of the non-structural genes of this novel G26P[19] strain provided additional evidence of associations between this virus and the corresponding segments of porcine and porcine–human reassortant strains (Tables 1 and S3, Fig. S2).

Taken together, our results suggested that the reported novel RV strain may have arisen from reassortment events involving porcine and porcine-like human strains in unidentified host(s). Owing to the nature of segmented viruses, the individual gene segments of this G26P[19] strain may have originated from several independent parental strains, as summarized in Fig. 2. The genome constellation of G26P[19] is perhaps most similar to the porcine-origin human strains RVA/Human-wt/THA/Mc345/1989/G9P[19] and RVA/Human-wt/THA/Mc323/1989/G9P[19] (Ghosh *et al.*, 2012), with 10 out of 11 genes belonging to the same genotype (P[19]–I5–R1–C1–M1–A8–N1–T1–E1–H1) (Table 1) and sequences of cognate segments often showing close phylogenetic association. We speculate that this G26P[19] strain might have arisen from reassortment events involving a G26 virus (possibly porcine in origin) and a virus similar to the aforementioned Thai G9P[19] strains, although the limited data available prevent us from investigating where, when or in what species this event may have occurred. Whilst a G26P[19] human RV was mis-identified as a novel G3P[19] in Thailand in 2004–2006 (Theamboonlers *et al.*, 2008), no other G26P[19] has been detected in subsequent RV studies in Thailand or in any continual RV surveillance programmes elsewhere. The detection of such similar viruses in two countries in South-east Asia raises questions as to the prevalence of this strain in human and animal RV infections, and the ability of this rarely detected strain to infect and transmit between humans. This warrants further epidemiological and biological investigation.

**Fig. 2.**
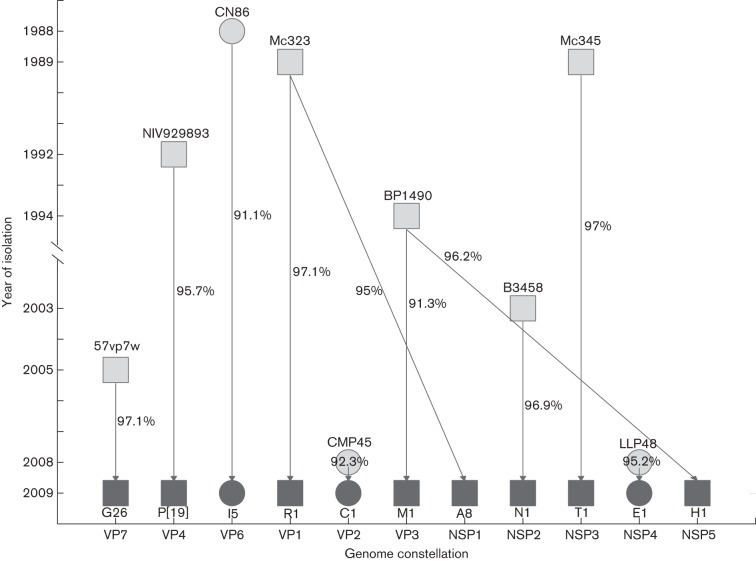
Schematic tracing of reassortment possibilities for the emergence of the G26P[19] strain by closest genetic distance. The graph illustrates the potential parental segments from strains that may have been involved in reassortment events giving rise to the G26P[19] strain described in this study. The horizontal axis indicates the 11 gene segments and the vertical axis indicates the year of strain identification. Truncated strain names are indicated above the marker; squares indicate porcine-like human origin and circles indicate porcine origin. All segments of our G26P[19] strain (in dark grey) are marked as a circle or square according to the host origin of its corresponding most closely related segment by genetic distance, which is given as the percentage nucleotide similarity next to arrows.

Given the potential for mixed RV infections, atypical strain circulation, and the close proximity of people and domesticated animals in parts of Asia, the characterization of genetic reassortment and interspecies transmission is key to understanding the evolutionary and ecological dynamics of RV (Cook *et al.*, 2004; Santos & Hoshino, 2005). Here, we have identified and described the genome of a novel G26P[19] RV in children with diarrhoea in southern Vietnam, confirming the circulation of potentially zoonotic RV strains alongside common human RV strains causing human disease. There is a lack of whole RV genome sequences available from this region of Asia (from both humans and animals), which limits our understanding of the epidemiology and zoonotic potential of this important human pathogen. Additionally, the efficacy of currently available vaccines [Rotarix (GlaxoSmithKline) and RotaTeq (Merck)] against novel and exotic RV genotypes has not, as yet, been determined. Continual RV strain surveillance, combined with whole-genome sequencing from both human and animal populations, is essential for understanding the epidemiological relevance of novel RV strains and assessing their zoonotic potential. Such investigations are essential for determining the potential impact of vaccination in urban and rural areas of Asia with alternative circulating organisms and atypical RV epidemiology.
